# Laponite^®^—From Dispersion to Gel—Structure, Properties, and Applications

**DOI:** 10.3390/molecules29122823

**Published:** 2024-06-13

**Authors:** Cristina-Eliza Brunchi, Simona Morariu

**Affiliations:** “Petru Poni” Institute of Macromolecular Chemistry, Grigore Ghica Voda Alley 41A, 700487 Iasi, Romania; brunchic@icmpp.ro

**Keywords:** Laponite^®^, dispersion, gel, drilling fluid, drug delivery, tissue engineering, cosmetics

## Abstract

Laponite^®^ (LAP) is an intensively studied synthetic clay due to the versatility given by its layered structure, which makes it usable in various applications. This review describes the multifaceted properties and applications of LAP in aqueous dispersions and gel systems. The first sections of the review discuss the LAP structure and the interactions between clay discs in an aqueous medium under different conditions (such as ionic strength, pH, temperature, and the addition of polymers) in order to understand the function of clay in tailoring the properties of the designed material. Additionally, the review explores the aging phenomenon characteristic of LAP aqueous dispersions as well as the development of shake-gels by incorporating LAP. The second part shows the most recent studies on materials containing LAP with possible applicability in the drilling industry, cosmetics or care products industry, and biomedical fields. By elucidating the remarkable versatility and ease of integration of LAP into various matrices, this review underscores its significance as a key ingredient for the creation of next-generation materials with tailored functionalities.

## 1. Introduction

Laponite^®^ (LAP) has attracted considerable interest among researchers due to its distinct structure, which confers remarkable colloidal stability in aqueous media. This characteristic makes LAP suitable for various applications, from rheology modifiers to stabilizers in emulsions and suspensions. The exceptional colloidal stability exhibited by LAP, arising from its unique structural attributes, underlines its versatility and utility in various industrial and scientific fields.

Generally, the dispersion of clay particles in water involves the establishment of interactions between the charged surface of the nanoparticles and the surrounding solvent molecules. Variations in pH and ionic strength, as well as the addition of additives (e.g., polymers, drugs, etc.), modify the stability and rheological properties of the dispersion, which are essential for their specific applications [1,2,3,4,5,6]. For example, in the presence of salts, the electrostatic repulsion interactions between the LAP nanoparticles in the dispersion are screened by the salt counterions [7]. Moreover, the interparticle interactions of LAP platelets and their aggregation are inhibited by the introduction in the clay dispersion of macromolecular chains [8,9].

The properties of LAP aqueous dispersion, including thixotropy, shear thinning behavior, and excellent film-forming capabilities, make LAP a valuable additive in areas such as cosmetics, pharmaceuticals, agriculture, and environmental remediation. In the field of drilling fluids, LAP is utilized for its ability to improve the stability and performance of drilling muds in oil and gas exploration operations [10]. The colloidal stability and biocompatibility of LAP are exploited in the medical field where it represents an ideal candidate for the formulation of controlled drug release systems [11]. Also, the large surface area and tunable surface chemistry of LAP make it a versatile platform for the fabrication of sensitive and selective sensing devices [12].

Understanding the behavior of LAP in aqueous dispersion and its response to various factors is essential for harnessing its full potential in diverse applications. This review aims to deepen the knowledge of the behavior of LAP in water, exploring the impact of factors such as the presence of salt or polymer, pH, time, etc., on its dispersion characteristics and elucidating its diverse applications across various industries.

## 2. Laponite^®^ Structure

In recent years, smectite clays have been intensively studied due to the interesting properties exhibited by them in the materials in which they are included [13]. The first Laponite^®^ (LAP) material was synthesized and patented in 1962 by Neumann B.S., a research scientist at Laporte Industries Limited, London, UK [14]. Since then, several types of LAP have been synthesized to improve the performance of the products in which they are included, giving them properties that correspond to the users’ requirements. Table 1 shows the most studied and used clays in the formulation of materials applicable in various fields. In addition to the variants of LAP mentioned in Table 1, new sorts of LAP were developed due to the need to tailor the properties of materials to specific applications. For example, Laponite^®^ S482, Laponite^®^ B, Laponite^®^ SL25, and Laponite^®^ RDS–P were prepared for the paper industry, ceramics, care products, and pharmaceuticals or cosmetics, respectively.

**Figure 1:**
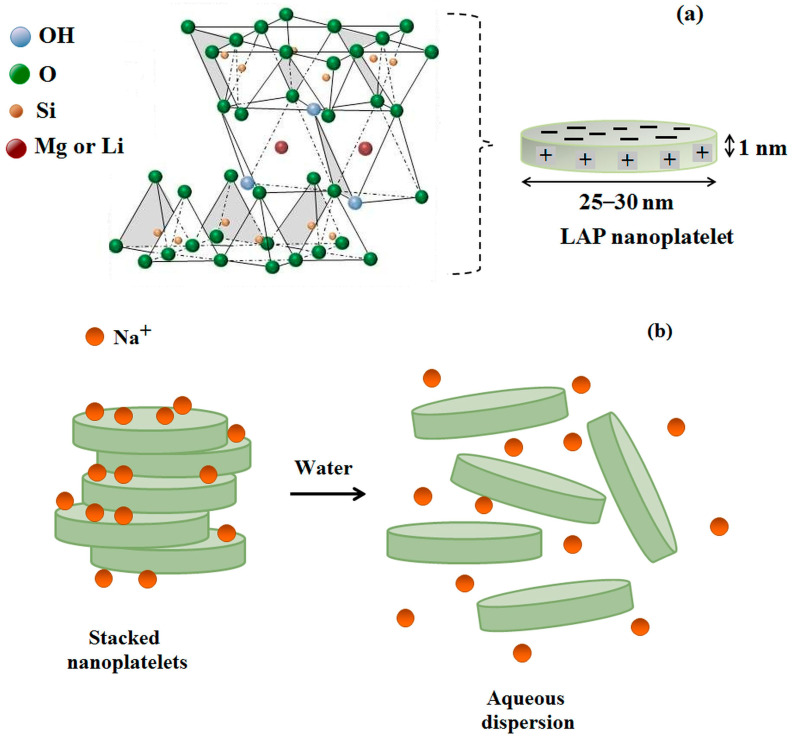
Schematic representation of the following: (**a**) Single LAP nanoplatelet; (**b**) Nanoplatelets exfoliation in water.

All LAP types have a similar base structure with the chemical formula Na^+^_0.7_[(Si_8_Mg_5.5_Li_0.3_)O_20_(OH)_4_]^−0.7^. LAP has a crystal structure characteristic of 2:1 phyllosilicates, in which a magnesium octahedral sheet is sandwiched between two silicon tetrahedron sheets (Figure 1a). In this structure, some magnesium ions (Mg^2+^) are replaced with lithium (Li^+^), causing the appearance of a negative charge on the platelet surface. This crystal has a disc-like shape with a diameter and thickness of about 25–30 nm and 1 nm, respectively [15]. A single platelet has about 1100 ± 100 unit cells and a molecular weight of 7 × 10^5^~9 × 10^5^ g/mol [16,17]. In a dry state, LAP discs are stacked, with their negative charges balanced by the Na^+^ ions between interlayers (Figure 1b). LAP nanoplatelets from these stacks disperse in water, and Na^+^ ions dissociate, inducing a strong negative charge on their surface. On the edge of platelets, predominately, are the MgOH groups, which acquire a weak positive charge at low pH due to the gain of hydrogen ions or a negative one due to the loss of hydrogen ions at high pH [18]. Tawari et al. [19] reported that platelet edge charges are neutralized at pH ≈ 11 (the point of zero charge, PZC).

In LAP dispersions with concentrations below 2% and pH < 9, the dissolution of clay particles, according to the following equation, was observed by Thompson and Butterworth [20]:

Na_0.7_[(Si_8_Mg_5.5_Li_0.3_)O_20_(OH)_4_] + 12H^+^ + 8H_2_O

↓

0.7Na^+^ + 8Si(OH)_4_ + 5.5Mg^2+^ + 0.3Li^+^

Mg^2+^ ions were also observed in LAP dispersion with a low clay concentration for a pH above 10 [1].

**Figure 2:**
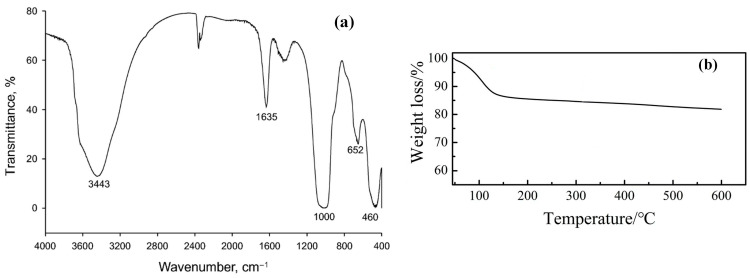
(**a**) FTIR spectrum (Reproduced with permission from [22]. Copyright © 2019, The Author(s)). (**b**) TGA curve of LAP (Adapted with permission from [23]. Copyright © 2019, Springer Nature Switzerland AG, Cham, Switzerland).

The FTIR spectrum (Figure 2a) shows several characteristic bands corresponding to specific molecular vibrations within the LAP structure. The broad band at 3443 cm^−1^ is attributed to the stretching vibrations of OH groups from the water molecules and hydroxyl ions adsorbed onto the clay surface. Another significant peak is observed around 1630–1650 cm^−1^, which also corresponds to the bending vibrations of water molecules adsorbed onto the clay surface. This band is indicative of the interaction between water and clay. The peaks at 460, 652, and 1000 cm^−1^ are assigned to Si–O–Mg, Mg–OH–Mg, and Si–O bending vibrations, respectively [21].

Thermogravimetric analysis of LAP (Figure 2b) shows its excellent thermal stability, losing only 9.7% of its mass before 113 °C due to the evaporation of interlaminar free water [23].

## 3. Laponite^®^ Aqueous Dispersion—Effect of Various Factors on the Clay Platelets Organization

### 3.1. Effect of Environment Ionic Strength and LAP Concentration

**Figure 3:**
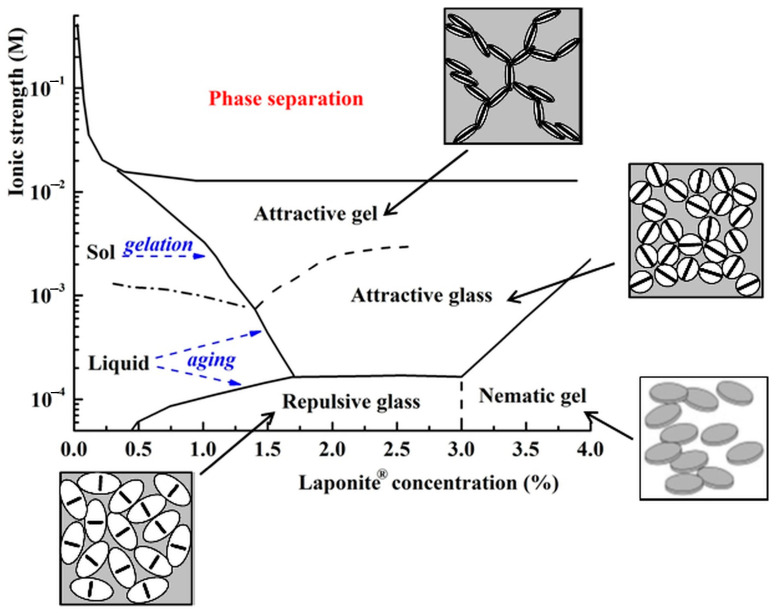
Phase diagram of LAP aqueous dispersion as a function of clay concentration and environmental ionic strength. In squares, the structures of each state are schematically represented (Adapted with permission from [2]. Copyright © 2004, American Physical Society, College Park, MD, USA).

The dispersions containing LAP revealed a complex state diagram in which particles can have various structural organizations. The phase diagram of LAP in water is still under debate due to its complexity given the possible multiple states, including liquid, sol, gel, glass, and flocculation (phase separation) [2,24,25,26]. In Figure 3, a phase diagram is presented as a function of LAP concentration and environmental ionic strength, which considers the reported scientific data [2,24,27,28,29,30].

At low LAP content, the dispersions are fluids, and at high LAP concentrations, the dispersions can form repulsive colloidal glass (stabilized by long-range repulsion) and attractive gel (resulting from van der Waals attraction interactions) phases. The two physical states differ by the platelets’ organization, which implies the existence of a fractal network in gel, unlike disordered organization in repulsive colloidal glass [31]. LAP dispersions can exhibit various disordered nonergodic states classified as attractive gel, characterized by dominated attractive interactions (a percolated network is formed), attractive glass (the attractive interactions influence the spatial distribution, but repulsive interactions still have a predominant role in the system dynamics), and repulsive glass (also called Wigner glass), in which the long-range electrostatic repulsions dominate [2,32]. In a nematic gel, most clay platelets are oriented in the same direction, but they can diffuse in all directions [33]. At LAP concentrations lower than about 1%, in the liquid phase, two different ergodic liquid states, sol, and liquid, were observed [2]. The liquid state is a homogeneous phase, while the sol state is inhomogeneous due to the presence of some clusters characterized by a finite lifetime. Therefore, phase transitions of LAP dispersions, induced by changing the concentration of clay or salt, are possible both within a regime (for example, in the ergodic regime—transition from liquid to sol or in the nonergodic regime—from repulsive glass to attractive glass or attractive gel.) as well as between the two regimes (from an ergotic phase to a nonergotic one and vice versa). The transitions from liquid to glasses and from sol to attractive gel are named “aging” and “gelation”, respectively. Although both transitions lead to a nonergodic state characterized by elasticity, their physical processes are different.

With salt added, the concentration of cations in the clay dispersion increases, and the electrostatic repulsion interactions between the negative charges on the clay surface are screened. Consequently, the interactions between LAP platelets change from repulsive to attractive, modifying the dispersion structure.

**Figure 4:**
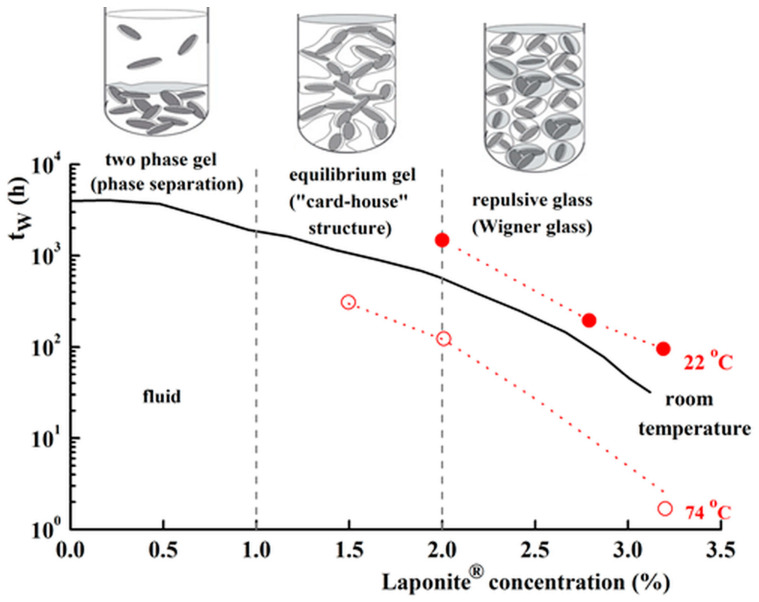
Effect of LAP concentration and temperature on the waiting time, t_w_. The solid black line corresponds to data from [34]; the red-filled and open symbols correspond to values from [35] (Adapted with permission from [34]. Copyright © 2010, Springer Nature Limited, London, UK).

For LAP aqueous dispersions, the rest time is an important factor when comparing various experimental data reported in the literature. The time to reach the equilibrium state (often referred to as aging time), t_w_, decreases with increasing LAP concentration and temperature (Figure 4) [34,35]. The aging phenomenon of LAP dispersions in the presence/absence of salt was investigated using rheological measurements [36,37,38,39,40,41], light scattering techniques [42], or ^23^Na Nuclear Magnetic Resonance [43,44]. The clay platelets undergo a structural rearrangement in time to attain a lower energy state.

The dispersions, with the LAP concentration lower than 2%, have aging times of the order of months or years, after which they exhibit two physical states: (i) For c_LAP_ ≤ 1%, a gel containing both clay poor domains (soil states) and clay-rich domains (gel states); (ii) For 1% < c_LAP_ < 2%, an equilibrium gel state with a “card–house” structure [34,35].

### 3.2. Effect of Polymer Addition

The addition of poly(ethylene oxide) (PEO) to LAP aqueous dispersions was intensively studied to clarify both the adsorption mechanism of the polymer on the LAP platelets and the effect of the molecular weight or concentration of the polymer on the dispersion state or properties of the LAP [45,46,47,48,49,50,51,52,53,54,55,56,57]. Although several adsorption mechanisms have been proposed, this subject is not fully elucidated. Most researchers consider that the mechanism of PEO adsorption on LAP platelets is similar to the adsorption of PEO on oxide surfaces, as proposed by Mathur and Moudgil [45]. They reported that silanol (SiOH) groups on silica (SiO_2_) surfaces can function as proton donors. The SiOH groups, formed on the disc surface due to some defects in the LAP structure, work like proton donators, and LAP behaves like a Brønsted acid. On the other side, PEO should be considered a Lewis base due to the electrons from the ether groups that can be donated. Moreover, the interaction between PEO (ether groups) and the clay surface (SiOH groups) can be seen as an interaction between a Brønsted acid and a Lewis base. Su and Shen [46] reported that PEO chains are adsorbed by hydrophobic interactions between –CH_2_–CH_2_– groups from the polymer and the siloxane surface of LAP.

The thickness of the PEO layer adsorbed was determined by small-angle neutron scattering measurements as being about 1.5 nm on each face and between 1.5 nm and 4.5 nm on the platelet edge for polymer molecular weights between 2 × 10^4^ and 9.65 × 10^5^ g/mol [47]. Excluding the sample containing PEO of 2 × 10^4^ g/mol, the thickness of the edge layer increases according to the following power–law dependence: the thickness of the edge layer ~M_w_^0.13^. The face layer thickness is independent of the added PEO molecular weight.

However, both polymer molecular weight and concentration can change the nature of interactions (attractive or repulsive) between LAP nanoplatelets, leading to various physical states. For 2% LAP dispersion, a critical molecular weight of PEO of 8.3 × 10^4^ g/mol, which limits two different behaviors (irrespective of pH and PEO concentration), was determined: (i) For M_W_ lower than this critical value, gelation kinetics slowing occurs; this behavior is explained by the steric barrier in the case of attractive gel or by the depletion attraction between clay particles in the case of repulsive glass, caused by the adsorption of the PEO chain; (ii) For M_W_ above the critical molecular weight, accelerated gelation takes place due to the polymer chain length, which is able to bridge the clay particles [48]. The increase in LAP concentration to 2.5% decreases the critical M_W_ value to about 2.5 × 10^4^ g/mol due to the increased possibility of bridging the clay particles by decreasing the distance between them [49]. 

A transition from the repulsive glass state to the liquid phase and then back to the attractive glass state (re-entrant behavior) was observed for the PEO/LAP dispersions with a fixed clay concentration [50,51]. 

**Figure 5:**
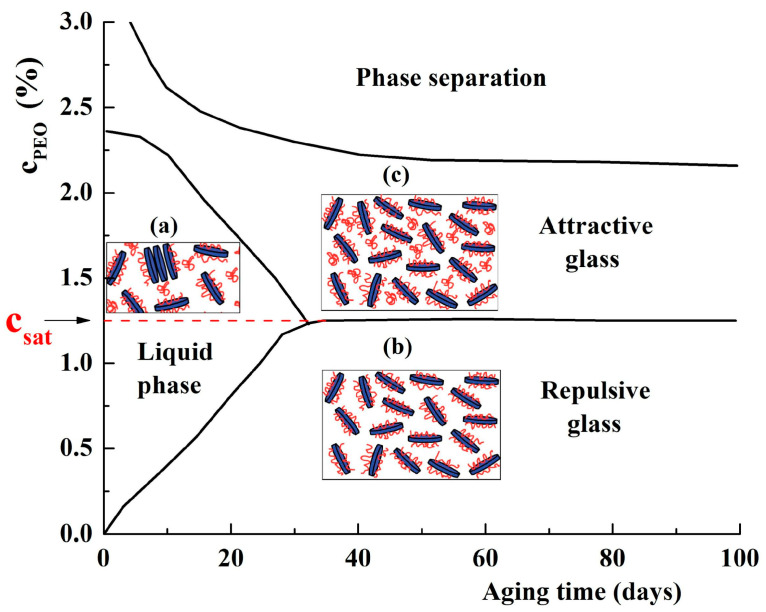
Phase diagram of 2% LAP dispersion as a function of PEO (Mw = 2 × 10^4^ g/mol) concentration and aging time. The inset figures schematically show the structure of LAP dispersions in the presence of PEO in (**a**) liquid state; (**b**) repulsive glass; and (**c**) attractive glass (Adapted from [51] with permission from the Royal Society of Chemistry, London, UK).

For 2% LAP dispersions at pH = 10, Atmuri et al. [51] reported a transition from a repulsive colloidal glass to a viscous fluid by the addition of PEO with a molecular weight of 2 × 10^4^ g/mol. After a rest period, which depends on the added polymer concentration, a glassy phase is reformed. A critical polymer concentration (c_sat_), corresponding to a complete cover of the clay platelet surface, was evidenced. By aging, the dispersion state changes from that of ergodic liquid to repulsive or attractive glass if the polymer concentration is below or above c_sat_ (Figure 5). Some clusters of LAP, the free polymer chains, and the adsorbed chains on the particle surface are present in aqueous dispersion without salt (inset (a) in Figure 5). Below c_sat_, the presence of free polymer chains slows down the rearrangement of clay particles and delays the transition to the repulsive glass phase (schematically represented in inset (b) in Figure 5). For the LAP dispersions containing PEO above c_sat_, after a long time, an attractive glass with some not adsorbed polymer chains is obtained (schematically represented in inset (c) in Figure 5).

**Figure 6:**
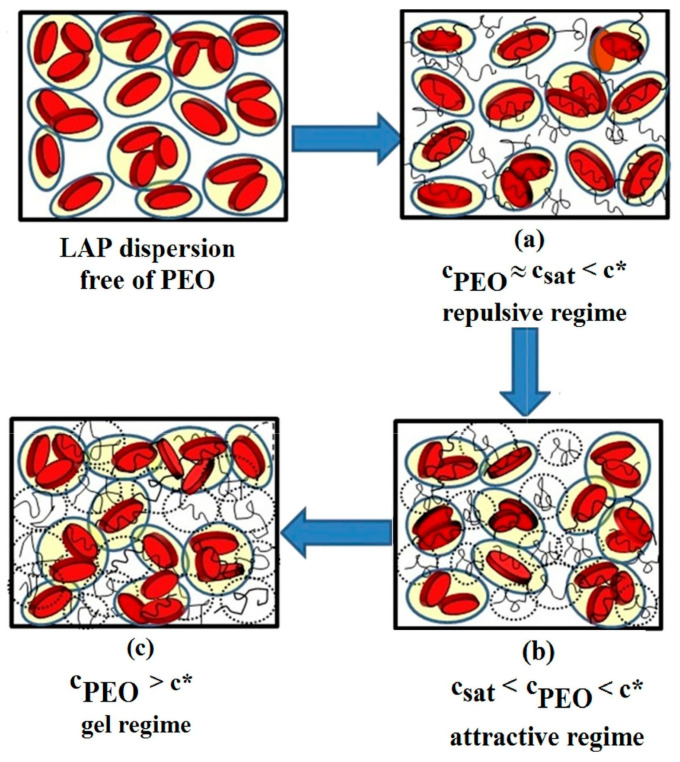
Schematic representation of the dynamics of clay particles and PEO chains in aqueous dispersion as a function of polymer concentration; (**a**) repulsive regime; (**b**) attractive regime; and (**c**) gel regime (Adapted with permission from [53]. Copyright © 2015 Wiley Periodicals, Inc., Hoboken, NJ, USA).

By adding PEO with a high molecular weight (3 × 10^5^ g/mol) to LAP dispersions with clay concentrations between 1% and 1.88%, Can and Okay [52] evidenced four physical states, namely, liquid, viscous liquid, weak, and strong gels. Besides c_sat_, the overlap concentration of PEO (c*) represents another factor involved in the structural organization of LAP dispersions [49]. With the addition of polymer, the interactions between clay particles are changed, leading to their structural reorganization due to the newly developed polymer–polymer and polymer–clay interactions (Figure 6) [53,54].

If the polymer concentration (c_PEO_) is around c_sat_ but lower than c*, the polymer chains adsorbed on clay particles increase the attractive interparticle interactions, leading to the repulsive glass state of clay dispersion (Figure 6a). For polymer concentrations between c_sat_ and c*, some small clusters, which can quickly diffuse in a dilute medium, are formed via short-range attractive interactions (Figure 6b). A further increase in polymer concentration intensifies the polymer–polymer interactions, limiting the motion of clay clusters in the system as it approaches c*. At polymer concentrations higher than c*, the packed polymer chains behave as a uniform polymer matrix (Figure 6c). The clay clusters and polymer matrix undergo structural modification as a result of competition between polymer–polymer and polymer–clay particle interactions.

The effect of various factors (i.e., polymer molecular weight, temperature, clay concentration) on the aging dynamics of PEO–LAP mixtures was investigated by dynamic light scattering measurements [55,56] or rheology [41,57].

### 3.3. Effect of pH

Most investigations on aqueous dispersions of LAP were carried out at high pH (pH = 10) and low ionic strength. Au et al. [58] showed that LAP platelets in dispersion have a negative charge in the range of pH between 3 and 12. The interaction type (attractive or repulsive) between LAP platelets was discussed, considering the zeta potential and yield stress values [58]. Moreover, the increase in pH from 8 to 12 determines the decrease in yield stress, which is correlated with an increase in the negative value of zeta potential. At low pH, the yield stress is higher due to the agglomeration of clay particles (low zeta potential), while at high pH, the yield stress value is lower because repulsive interactions are predominant in the LAP dispersion. Moreover, 1% LAP dispersion exhibits a zeta potential of −62.5 mV (at pH = 10) as a result of the repulsive forces that are dominated in the dispersion [58]. 

Below the point of zero charge (PZC) (pH = 11), the edges of LAP particles are positively charged, while above that, they become negatively charged. This charge variation is due to the dissociation of H^+^ ions above the PZC and OH^−^ ions below the PZC. Consequently, when H^+^ ions dissociate above pH = 11, the pH of the dispersion decreases, and when OH^−^ ions dissociate below pH = 11, the pH increases (as it was also mentioned in the section concerning LAP structure). These changes in pH significantly affect the stability of the LAP dispersion. Jatav and Joshi [1] investigated the stability of LAP in aqueous dispersion, focusing on the initial pH of the water and the LAP concentration. The dissolution of LAP is assessed by measuring the leached magnesium concentration. The initial pH of water, between 3 and 10, does not impact LAP dissolution. LAP dissolves even at a pH above 10 when its concentration is low (1 to 1.7%). However, at higher concentrations (2.8%) and similar pH values, no dissolution is observed. The Na^+^ concentration increases in dispersion by increasing LAP concentration, and they could give greater stability to the LAP dispersions [1].

### 3.4. Effect of Temperature

Temperature can have a significant impact on the viscosity and stability of LAP dispersions, and understanding the effect of temperature on the LAP dispersion properties is crucial for optimizing its performance in various applications. LAP exhibits exceptional thickening ability and thermal stability, maintaining its viscosity even at temperatures as high as 260 °C [23]. In a 4% bentonite dispersion mixed with 1% LAP, the viscosity remains consistent before and after aging at 260 °C for 16 h, exhibiting its thermal resilience. Compared to other high temperature viscosifiers, LAP demonstrates superior performance even at ultrahigh temperatures. These qualities make LAP suitable for formulating ultrahigh temperature water-based drilling fluids [23]. Awasthi and Joshi [59] evidenced that the aging of LAP dispersions becomes faster at higher temperatures, thereby shifting the evolution of storage modulus to a lower age at higher temperatures. The additives, such as salts and polymers, can also interact with LAP in dispersion differently at varying temperatures, further impacting its rheological properties. Moreover, for aqueous dispersions with 2% poly(ethylene glycol) (PEG) and LAP concentrations exceeding 3%, the effect of temperature on the longest relaxation time becomes more important [41]. This behavior is likely attributable to the impact of Brownian motion on the formation of a robust network structure, along with changes in the solubility of PEG in water at higher temperatures, especially notable in mixtures with higher clay concentrations across various temperature ranges. The gelation time of the LAP aqueous dispersions containing 2% PEG decreases from 1 week to 2 h when the clay concentration increases from 2% to 4% [41].

## 4. Properties of Laponite^®^ Aqueous Dispersions and Hydrogels

### 4.1. Rheological Properties

**Figure 7:**
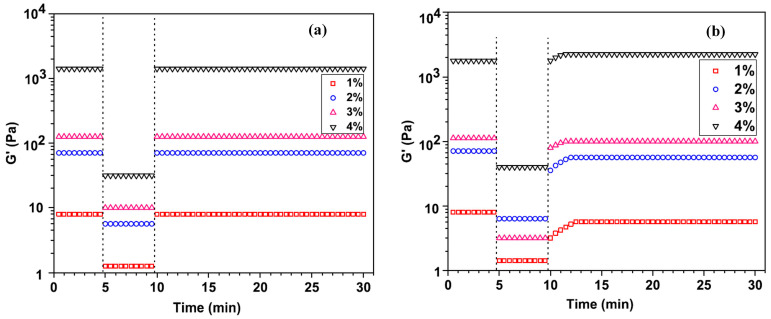
Effect of LAP concentration on the thixotropy of (**a**) LAP dispersion and (**b**) chemically crosslinked LAP/PAAm hydrogel (components 5% AAm, 0.03% K_2_S_2_O_8_, 0.015% N,N′–methylenebis acrylamide) (Reproduced with permission from [62]. Copyright © 2021 Elsevier B.V. (Amsterdam, The Netherlands) All rights reserved).

Knowing and understanding the phase diagram of the LAP dispersion allows the adaptation of the working conditions in order to obtain the desired physical state and, implicitly, the required property of a specific application. LAP separation at pH = 10 could be possible if two conditions are ensured: (i) A high amount of monovalent salt to be added in order to screen the repulsive interactions between clay platelets; (ii) A neutral polymer such as PEO to be added to bridge the clay platelets [60]. By adding polymer and salt simultaneously to LAP aqueous dispersions, platelet–platelet separation is the result of the competition between two mechanisms: attractive ion–ion correlation and polymer bridging [61]. It is well known that due to the arrangement of clay particles in a network structure, LAP dispersions exhibit strong shear thinning and thixotropic behavior (Figure 7a) [62]. A thixotropic hydrogel for water shutoff in horizontal wells was developed by adding LAP nanoparticles to an acrylamide solution subjected to chemical crosslinking. The increase in concentration of LAP enhanced the thixotropic performances (Figure 7b) [62]. Moreover, the polymer systems in which LAP is included acquire these properties depending on the concentration of the components and the structure of the polymer. The improvement of the thixotropy of a material is essential for its use in some applications, such as drug delivery, tissue engineering, bioprinting, drilling fluids, cosmetics, etc. The addition of carboxymethyl cellulose (CMC) in an LAP dispersion reinforces the network and leads to a gel with strong shear thinning and thixotropy [9]. The thixotropic and shear thinning behavior of CMC/LAP gels is due to the physical interactions between OH and SiO groups from CMC and LAP, respectively, which strengthen the gel network. The effect of the addition of an ionic monomer, such as 2-acrylamido-2-methylpropanesulfonic acid (AMPS), on the stability and rheological properties of LAP dispersion was investigated by Li and coworkers [63]. The addition of AMPS in concentrations up to 2% in LAP aqueous dispersion causes the aggregation of LAP particles. In the presence of this ionic monomer, the electrostatic repulsions between clay particles decrease while the van der Waals attraction forces increase. At a concentration above 2%, AMPS determines the chemical degradation of LAP under acid attack. The rheological measurements showed that LAP/AMPS aqueous dispersion passes from pseudoplastic fluid to Newtonian fluid by increasing the ionic monomer concentration.

Hydrogels with superior rheological properties were obtained by Sebenik et al. [64] by adding LAP nanoparticles to a network based on tempo–oxidized cellulose. For low LAP concentrations, the clay acts as a bridging agent between polymer nanofibrils, increasing the elastic properties of the systems. In systems containing higher amounts of LAP, the edge–face interactions between clay platelets are predominant, giving a gel-like structure in which the contribution of polymer nanofibrils becomes less important. In contrast, for LAP/scleroglucan hydrogels, the rheological parameters increased with the increase in clay concentration [65]. The different behavior of the two hydrogels can be attributed to the polymer component structure. A small amount of clay platelets in LAP/scleroglucan hydrogels is not able to form a network by bridging the polymer chains due to the nonionic nature of scleroglucan.

**Figure 8:**
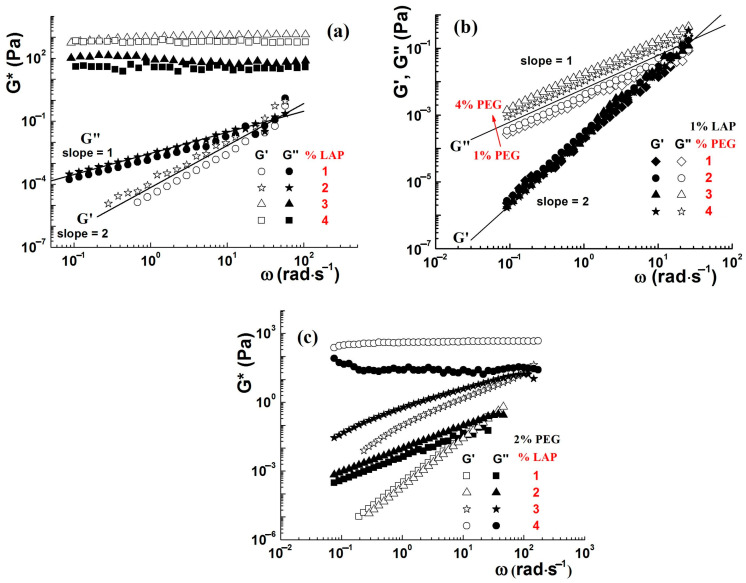
Oscillatory frequency dependence of the viscoelastic moduli for the following: (**a**) LAP dispersions with various clay concentrations; (**b**) 1% LAP dispersion with different PEG concentrations; (**c**) 2% PEG with different LAP concentrations ((**a**,**c**) Adapted with permission from [41]. Copyright © 2012, American Chemical Society (Washington, DC, USA) and (**b**) Adapted with permission from [66]. Copyright © 2009, American Chemical Society).

The aqueous dispersions of LAP without polymers exhibit various rheological behaviors as a function of clay concentration. Moreover, the dispersions containing less than 2% have liquid-like behavior, while those with higher LAP content show solid-like behavior (Figure 8a) [41]. The addition of PEG (M_W_ = 10^4^ g/mol) up to 4% does not significantly change the viscoelastic properties of 1% LAP dispersion (Figure 8b) [66]. The variation of storage (G′) and loss (G″) moduli with the oscillation frequency is typically for a Maxwellian fluid, with G″~ω^1^ and G′~ω^2^. The increase in PEG amount determines a slight increase in loss modulus due to the breaking of the network formed by the clay platelets and the shielding of the interactions between them [66]. The short PEG chains are adsorbed on the clay particle surface, forming a steric barrier between clay platelets, and they do not allow the formation of interparticle bridges, and as a result, the value of G′ remains unchanged [48]. The effect of clay concentration on the rheological behavior of LAP/PEG dispersions is more significant. The enrichment of the mixture in clay determines the increase in viscoelastic moduli and the change in flow behavior from liquid-like to solid-like (Figure 8c) [41].

The elasticity of LAP/PEO gel can be improved by adding a small amount of chitosan (CS), which strengthens the gel network due to the formation of additional interactions between CS chains and clay platelets, or PEO chains [5,67].

It is well known that some clay dispersions containing PEO show an unusual nonNewtonian behavior manifested by the ability to form a gel when they are shaken. Under specific conditions, LAP dispersions can undergo the phenomenon of increase in viscosity by increasing the shear rate so that the dispersion can become a gel by simply shaking, making them potential candidates for the development of shock absorbers in various industries (i.e., the car industry). Liu et al. [68] showed that when the number of silica particles per one PEO chain (M_W_ = 2 × 10^6^ g/mol) exceeds 2, the mixtures exhibit a shear thickening behavior. Cabane et al. [69] also reported the shear thickening phenomenon for the silica particles/PEO mixture containing very long PEO macromolecules (M_W_ = 4 × 10^6^ g/mol) at compositions for which the silica particle surfaces are not completely covered with adsorbed polymer. They consider that, at rest, the particles associate into long necklaces due to the adsorption of each PEO chain on the few particles. By applying a shear, a partial desorption of polymer chains occurs, allowing the association of necklaces, which leads to the gelation of the system. At higher shear, these necklace associations collapse, forming the three-dimensional flocs. The shake-gel formation highly depends on the degree of particle surface coating with polymer, which in turn depends on the clay particles and PEO concentration, polymer molecular weight, particle shape, and size [70]. Moreover, the particle covering degree with polymers to form shake-gel is around the value corresponding to the complete covering for disk-like particles and around 2/3 of that limit for spherical particles. At some PEO concentrations, the shaking of dispersions containing the micrometer-sized particles of montmorillonite leads to an irreversible phase separation. In contrast, by shaking the dispersions containing nanometer-sized particles of LAP, a strong gel is formed.

The nature of the applied shear is another factor that influences gel formation. Moreover, in the case of LAP/PEO dispersions, the application of a shear rate up to 10^3^ 1/s does not lead to gel formation for many minutes, while for a manual agitation corresponding to a shear rate lower than 10^2^ l/s, the gelation occurs in a few seconds [71]. Under high shear, the formed clay aggregates are oriented, and the number of collisions between them decreases, preventing further aggregation even after a longer period of time. The shake gelation is reversible, and the systems become fluid again when the shear stops. After shear stops, the transition time from shake-gel back to the sol state (relaxation time) is dependent on the polymer concentration. Hence, the relaxation times of a dispersion with 1.25% LAP and PEO with a molecular weight of 3 × 10^5^ g/mol decrease from approximately 8 × 10^4^ s to 2 × 10 s by increasing the polymer concentration from about 0.3% to 0.46% [72]. With the increased polymer concentration, the clay particle surfaces are completely covered, the shear favors the formation of new bonds, and the relaxation time decreases.

The formation of shake-gels was discussed by Can and Okay [52] for LAP/PEO aqueous dispersions in terms of the average number of PEO chains adsorbed on a LAP particle, ${\bar{n}}_{PEO}$. For PEO with M_W_ = 3 × 10^5^ g/mol, the shake-gel formation was observed for ${\bar{n}}_{PEO}$ in the range of 0.1–0.2, and this range decreases with increasing PEO molecular weight. With the use of PEO with a molecular weight between 2 × 10^5^ g/mol and 3 × 10^5^ g/mol, strong gels with relaxation times higher than 30 min were obtained.

Recently, Huang, and Kobayashi [73] reported that the physical state of silica nanoparticles/PEO suspensions depends on pH, the PEO dose per silica surface area, and the PEO molecular weight. The shake-gel phenomenon occurs in the pH range of 8–9.4. Shake-gels with longer relaxation times were obtained by using PEO with molecular weights between 10^6^ and 4 × 10^6^ g/mol. They determined that the relaxation time increases with the increase in PEO molecular weight with a power–law relationship where the power is 3.38.

Under stress, two concurrent processes occur in the PEO/LAP aqueous dispersions: (i) The clay–clay and clay–polymer aggregates, formed at rest, are deformed and broken; (ii) Clay particles have a high probability of binding to other polymer chains in the system, forming new clay–polymer bridges. The increase in shear rate causes the breaking up of the aggregates as well as the extension of the polymer chains, which are adsorbed on several clay particles, leading to an increase in aggregate size and, consequently, an increase in the viscosity of the system [74].

**Figure 9:**
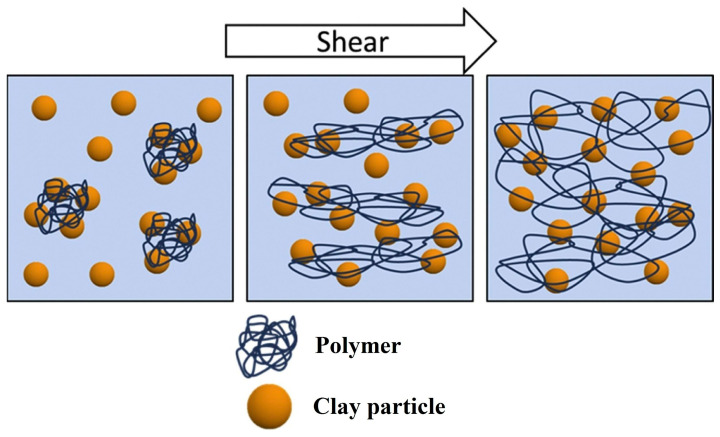
Schematic representation of the gelation mechanism under shear (Adapted with permission from [76]. Copyright © 2017 Elsevier B.V. All rights reserved).

The extension of the clay–polymer network under shear was evidenced by small-angle neutron scattering [75]. By increasing the shear rate, the clay discs orient first with their surface parallel to the vorticity direction, and then the polymer network starts to stretch. In the aqueous dispersion of clay and polymer with higher molecular weight, the polymer coils are adsorbed on the surface of several particles, forming small aggregates (Figure 9). Under shear, these aggregates are stretched and collide with others, and the polymer chains are subjected to a successive adsorption/desorption process, leading to an increase in the size of the aggregates. The formation of a more extensive network containing clay particles and polymer chains adsorbed on them could cause gelation and thickening under shear. By stopping the shearing, the polymer chains desorb from the surface of the clay particles, and the system returns to its initial liquid state [76]. Some rheological investigations have shown the existence of a critical shear rate at which a shear thickening transition occurs [74,77]. This critical shear rate value increases by increasing the polymer concentration. Below the critical shear rate, hydrodynamic forces are not able to break the micron-sized aggregates, and a mixture of these aggregates, free clay particles, and polymer chains coexist in the system. Above the critical shear rate, the micron-sized aggregates are broken, and some new clay–polymer bonds are formed. At rest, the system returns to a liquid state due to the relaxation of the network. The energy of the newly formed bonds is a few k_B_T, and a thermal fluctuation is sufficient for the system to return to its more stable initial configuration [72]. For silica/PEO, the adsorption energy was reported to be about 1.2 k_B_T per mol of segment [78].

Recently, shear thickening was also reported for some physical hydrogels based on LAP and poly(vinyl alcohol) (PVA) obtained by the freezing/thawing technique [4]. The hydrogels containing between 2% and 2.5% LAP and 4% PVA showed an increase in viscoelastic parameters by applying high deformation.

### 4.2. Mechanical Properties

**Figure 10:**
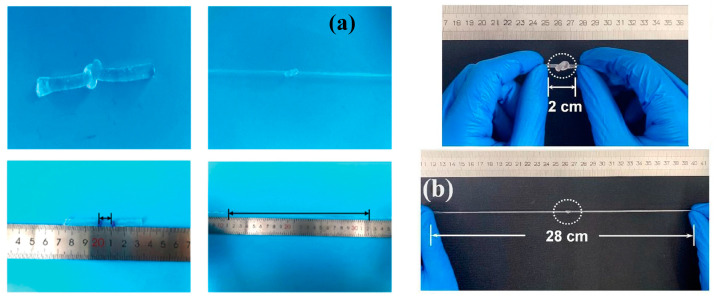
Mechanical properties of (**a**) LAP/PAA/PAAm and (**b**) LAP/PAA hydrogels ((**a**) Reproduced from [79] with permission from the Royal Society of Chemistry and (**b**) Reproduced with permission from [80]. Copyright © 2021 Elsevier Ltd. (Amsterdam, The Netherlands) All rights reserved).

The mechanical properties of polymer hydrogels are affected or improved by adding LAP, depending on both the structure of the polymer and the technique used in preparation. Generally, the ability of clay particles to form crosslinking points in the polymer networks (due to the adsorption of polymer chain segments on the surface of the clay particles) makes the simple addition of LAP to a polymer gel an improvement on its mechanical properties. Liu et al. [79] developed nanocomposite hydrogels based on LAP, poly(acrylic acid) (PAA), and poly(acrylamide) (PAAm) for enticing applications with excellent tensile strength, stretchability, and anti-fatigue properties. The hydrogel containing 4.5% PAA, 0.3% PAAm, and LAP/PAA (g/g) = 15 endured a very large stretching (strain > 2000%) without visible damage (Figure 10a). The increase in LAP concentration from 4.5% to 15% determined the increase in the tensile strength of the hydrogel from ~600 kPa to ~1.2 MPa [79].

The poly(acrylic acid) (PAA)/LAP hydrogels exhibited a maximum tensile stress of 1.18 MPa and a tensile strain of 2192%, which are significantly higher than the hydrogel without LAP, which has a tensile stress of 0.136 MPa and a tensile strain of 1223% (Figure 10b) [80]. This improvement is attributed to the physical crosslinking between LAP and PAA, which enhances the mechanical properties of the hydrogels. The heterogeneity of the bridge lengths between crosslinking points is responsible for the easier breaking of the chemically crosslinked PAA network. The PAA/LAP hydrogels have longer, more flexible chains due to uniformly distributed physical crosslinking points.

The tensile strength of the PVA hydrogels decreased significantly with the addition of LAP, from 10.82 MPa in clay-free PVA hydrogels to 2.05 MPa in those with 2.7% LAP [4]. This decrease was attributed to the formation of PVA crystalline zones during the freeze/thaw process and clay aggregates within the voids of the PVA network. The presence of clay aggregates reduced the interfacial area between PVA chains and clay nanoparticles, creating domains susceptible to mechanical stress [81]. The tensile modulus increased with LAP addition, from 1.63 MPa in PVA hydrogels to 2.56 MPa in the one containing 2.7% LAP, indicating enhanced stiffness. The disadvantage of reducing the mechanical properties of PVA hydrogels by incorporating an inorganic filler was solved by introducing multiple nanoscale minerals. Thus, the tensile strength and elongation at the break of the PVA hydrogel, which has incorporated LAP, layered double hydroxide, and hydroxyapatite could reach 16 MPa and 475%, respectively [82].

## 5. Laponite^®^ Applications

Laponite^®^ offers distinct advantages over natural clay, but it also comes with its own set of drawbacks (Table 2). Its advances lie in its uniform structure, high surface area, colloidal stability, and tunable properties. The uniform structure of LAP disc-like nanoparticles enables precise control over properties, making it suitable for various applications, ranging from biomedical to industrial. In addition, its high surface area facilitates interactions with other molecules, enhancing adsorption and reinforcement in composite materials. LAP exhibits exceptional colloidal stability, remaining dispersed in aqueous solutions without agglomeration or sedimentation. This stability is advantageous for applications requiring prolonged suspension or homogeneous dispersion. Furthermore, the tunable properties of LAP allow for customization to meet specific application requirements. By adjusting factors like pH, temperature, and additives, its rheological behavior, swelling capacity, and interlayer spacing can be tailored. However, despite its advances, LAP also presents some disadvantages. One significant drawback is its cost, as production processes can be more expensive than the extraction of natural clays. Additionally, its production process can have environmental implications, including energy consumption and waste generation. Moreover, the limited availability and specific applications of LAP can hinder its scalability and versatility.

**Figure 11:**
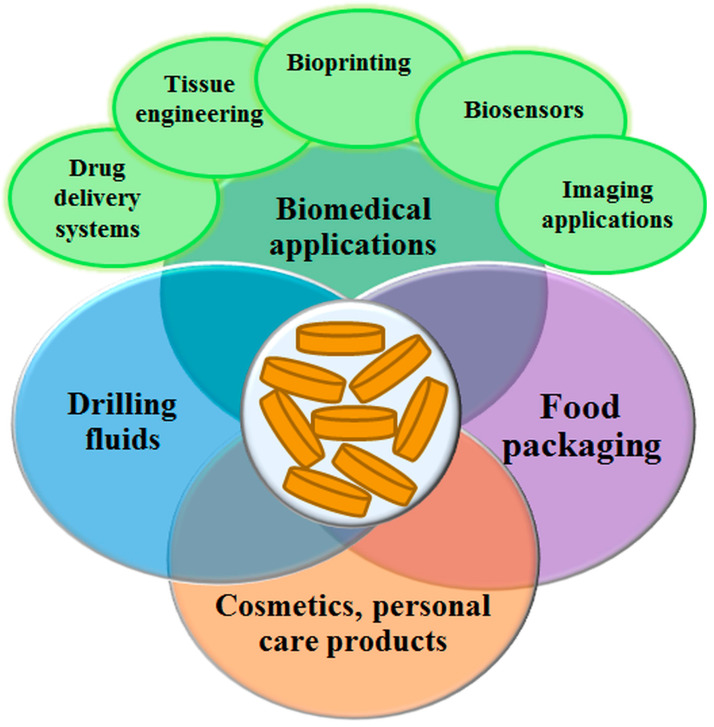
The main applications of Laponite^®^.

The properties of LAP, such as biocompatibility, the ability to exchange cations, to swell in water, to coagulate blood, to be shear thinning, thixotropic, stable over a wide range of pH and temperature, to have yield stress, antioxidant activity, a large surface area, etc., expand the range of its applications. Referring only to a limited number of works from the multitude of published ones, the most recent uses of LAP-based nanocomposite materials (in biomedical applications, drilling fluids, cosmetics, personal care products, and food packaging) as hydrogels, aqueous dispersions, or solid films will be briefly presented in the following (see Figure 11).

### 5.1. Biomedical Applications

#### 5.1.1. Drug Delivery

In the biomedical domain, hydrogels combine their ability to retain a large amount of water in their three-dimensional network with the softness, flexibility, biocompatibility, degradation, and cytotoxic behavior required for targeted applications. Recently, some reviews have extensively presented nanomaterials based on LAP that are attractive for the design of drug delivery systems [83,84,85]. Drug delivery systems based on hydrogels are widely used as vehicles to introduce a therapeutic substance into the body by ingestion, dermal dressing, or injection when, at room temperature, the hydrogel is fluid and, at body temperature, it becomes a gel. The improvement of these systems led to an increase in the effectiveness and safety of the drug by adjusting the time, location, and rate at which the therapeutic compound is released. LAP turned out to be an excellent candidate as a drug delivery carrier due to its aluminosilicate layered structure, which is able to incorporate different drugs (e.g., itraconazol [86], donepezil [87], ciprofloxacin [88], dexamethasone [89], microbial peptides [90], and simvastatin [91,92]) and control their release by ion exchange while also protecting and stabilizing fragile drugs or biomolecules.

**Figure 12:**
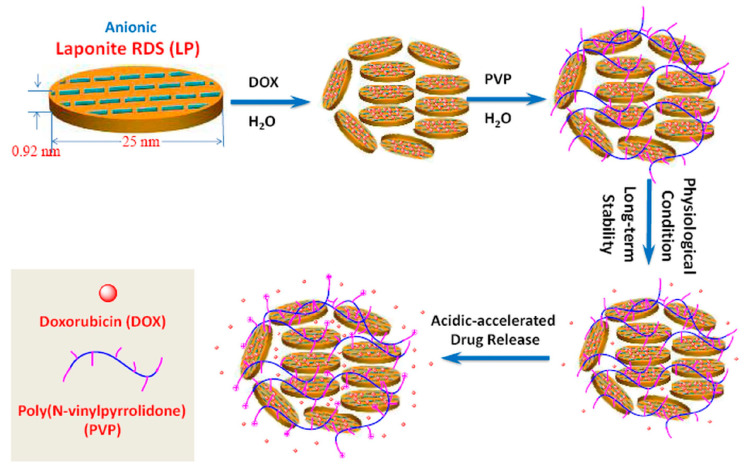
Schematic representation of the development path of LAP/PVP nanohybrids (Reproduced from [93] with permission from the Royal Society of Chemistry).

Wang et al. [93] have created multifunctional nanohybrids by self-assembling a pH sensitive poly(N–vinylpyrrolidone) (PVP) onto LAP without the use of an organic solvent. In these nanohybrids, the anticancer drug doxorubicin (DOX) can be encapsulated through electrostatic interactions with negatively charged LAP. The hydrophobic component of PVP binds to LAP, while its hydrophilic components form a protective shell, stabilizing the system. The ability of PVP to switch between protonated and deprotonated states gives the nanohybrids dual sensitivity to pH and temperature. In physiological conditions, these nanohybrids can remain stable over a long time and show enhanced DOX release in acidic extracellular environments, such as those found in solid tumors and endo/lysosomal compartments due to PVP protonation (Figure 12) [93].

A doxorubicin (DOX) delivery system based on LAP and cyclic poly(ethylene glycol) (cPEG) was developed by Tang and coworkers [94]. The LAP/cPEG/DOX formulation demonstrates higher drug loading efficiency compared to LAP/linear methoxy PEG/DOX. In phosphate–buffered saline, only 15% of DOX was released from LAP/cPEG/DOX, while in human plasma, approximately 90% of the drug was continuously released over 24 h. Additionally, the LAP/cPEG/DOX formulation, with a DOX concentration of 1–2 μM, exhibits enhanced inhibition of A549 lung carcinoma epithelial cells compared to the LAP/linear methoxy PEG/DOX system.

The hydrogel based on LAP as gelator and molecules of curcubit[6]uril (CB[6]) exhibits a very slow and sustained release of flufenamic acid (FFA) within 24 h at 37 °C and a pH of 5, which mimics the pH of the skin [95]. This is also confirmed by molecular modeling calculations; the release mechanism was explained considering that FFA released from the CB[6] cavity interacts first with the surface of the CB[6] and then spreads in the gel.

The LAP/chitosan (CS)/PVA hydrogels exhibit pH-responsive behavior, releasing 27.9% of curcumin in 12 h at pH 5.5 and 12.3% at pH 7.4, with cumulative releases of 48.5% and 18.5% over three days, respectively [11]. In vivo studies demonstrated lower cytotoxicity against cancer cells compared to free curcumin, confirming controlled drug release. Additionally, the hydrogels show good blood compatibility, antioxidant properties, and antibacterial activity, suggesting their potential application as pH-controlled drug delivery systems for cancer therapy.

Basu et al. [91] developed a nanocomposite hydrogel based on LAP, deoxyribonucleic acid (DNA), and oxidized alginate by chemical crosslinking of DNA (containing NH_2_ groups) and oxidized alginate (containing CHO groups). The reversible imine linkages formed are responsible for the shear thinning and self-healing properties of hydrogels. Additionally, physical crosslink points are created through the electrostatic interactions between LAP nanoplatelets and the negatively charged DNA. This formulated hydrogel demonstrates efficiency as a drug delivery vehicle for small molecules as an alternative to growth factors for stem cells (for example, simvastatin) or to promote bone tissue regeneration. Considering the antibacterial effects of simvastatin, the higher affinity for the epidermis than for the dermis [96,97], and the results obtained in the treatment of skin infections associated with melanoma [98,99], Suterio et al. [92] have prepared LAP-based gels containing lipophilic simvastatin, different LAP concentrations, and two skin permeation enhancers, namely isopropyl myristate (MYR) and squalene (SQ). The interaction of the drug and the permeation enhancer with the charged surfaces of the LAP in the hydroalcoholic medium leads to the formation of tactoids (ordered layered structures) with an impact on drug release. Also, the formulation with MYR exhibits better drug retention in the skin than that with SQ.

A soft and flexible hybrid nanohydrogel platform (with a value of elastic modulus ≈ 3 kPa) suitable for delivering antineoplastic drugs was tailored by Becher et al. [100]. The nanohydrogels containing LAP, poly(acrylate), and sodium phosphate can encapsulate more than one drug, which means a stronger action on cancer cells as a result of the synergistic effect of the drugs. Once the nanohydrogels are absorbed into the cell, they swell and release the antineoplastic drugs according to the edosomal pH.

The synergic action of heparin and DOX demonstrated the best antitumor efficacy in vitro and in vivo of the temperature sensitive injectable hydrogel, which consists of heparin–poloxamer 407 (HP copolymer) and LAP nanoparticles [101]. DOX molecules pre-adsorbed on the LAP nanoplatelets through ion exchange were then incorporated into the HP copolymer. Injectable hydrogels are widely used as biomaterials due to their smart features (tunable structures and stimuli-responsive biodegradation properties). Moreover, they have found utility in both the formulation of the delivery systems of the drugs or other active ingredients (proteins, genes, etc.) and in medical applications such as tissue engineering, regenerative medicine, bioprinting, aesthetic corrections, etc. [102,103,104].

#### 5.1.2. Tissue Engineering

In skeletal tissue engineering, LAP is one of the most commonly used nanoclays due to its ability to turn stem cells diverted from bone marrow into bone cells [105]. For such applications, LAP-based injectable hydrogels with multifunctional and self-healing properties have gained the attention of researchers [105,106]. The cell-laden hydrogels allowed not only their reorganization into tissue-like architectures but also the exchange of nutrients and wastes with the environment. A moldable, freestanding, and self-healable hydrogel was developed using a mixture of LAP and dendritic molecules [107,108]. The reversible interactions between the negatively charged LAP and those positively charged amine–terminated groups within the dendritic molecules lead to an injectable hydrogel with good healing (the healing time is less than 1 h) and shape memory properties (recovery is close to 100%). By using poly(carboxybetaine methacrylamide–co–hydroxyethyl methacrylate) mixed with LAP, the healing time of the obtained hydrogel has been significantly improved (i.e., reduced to 5 min) [109]. The shear thinning and thixotropic behavior of LAP have been used in the development of shear thinning hydrogels containing anionic alginate [106]. These hydrogels could be used in the removal of polyps and early–stage tumors through endoscopic resection of the mucosa. Results show that after 2 h of incubation, the alginate hydrogels mixed with LAP in different concentrations (between 1 and 3 mg/mL) maintain the initial heights of the submucosal cushion much better than saline solutions; this demonstrates their ability to be used as safe injectable solutions in endoscopy by making a durable submucosal cushion. In endoscopic applications, the hydrogels must overcome two resistances, namely, resistance to injection and resistance to dripping due to trauma. The use of endoscopy to deliver hydrogels directly to the surface of trauma or mucosal layers, aiming at hemostasis, wound protection, and facilitating healing, is a growing trend, along with the use of submucosal injections [110,111]. For endoscopic applications, the LAP hydrogel, free of other components, has also proven to be usable [104]. Ranjbardamghani et al. [112] developed a material for cartilage regeneration based on CS and LAP as components and a hybrid crosslinking method by using genipin and β-glycerophosphate. The obtained injectable hydrogels revealed improved elastic properties, compressive strength, antibacterial activity, and cell attachment significantly only for a content of 1% LAP. The hydrogen bonds between LAP and CS decrease the time and temperature of the gelation.

Nanocomposite hydrogels based on polyethylene–glycol diacrylate (PEGDA) and LAP were designed by Magalhães and coworkers [113]. The results from in vivo experiments on these hydrogels revealed significant bone regeneration, suggesting their potential application in orthopedics.

Recently, Rodrigo et al. [114] reviewed the latest data regarding the possible use of LAP in the preparation of materials potentially applicable in ophthalmology. Scientific research has shown that LAP can be included in all ocular structures and tissues due to its properties, such as biocompatibility, optical transparency, nanosize, thickness, and thixotropy, which facilitate easy injection and make it a candidate suitable for ophthalmological applications.

#### 5.1.3. Bioprinting

**Figure 13:**
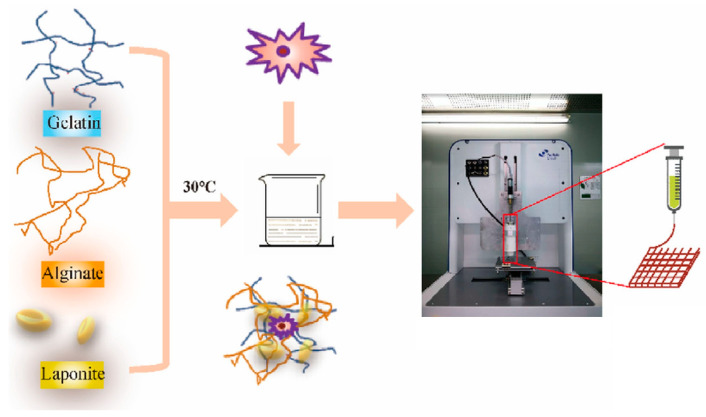
3D printed bioink based on LAP, gelatin, and alginate (Reproduced with permission from [120]. Copyright © 2022 The Authors. Published by Elsevier Ltd.).

Bioprinting is defined as the process by which living cells and biomaterials are arranged in a layered structure with a digital program, generating three-dimensional (3D) tissue-like architectures [115,116]. 3D bioprinting technology allows functional and efficient structures such as bones, tissues, and organs, including cartilage, skin, bones, and heart valves. Most bioprinting and biofabrication studies use alginate and gelatin for bioink formulations [117]. The incorporation of LAP in 3D printing improves the mechanical properties of hydrogels and cell adhesion [118] and promotes the transformation of cells toward osteoblastic differentiation [119]. Although LAP hydrogel alone cannot promote bone formation, Miao et al. [120] developed a 3D printed bioink based on LAP and loaded with bone marrow stromal cells for bone regeneration (Figure 13).

Aligned poly(caprolactone) microfiber scaffolds that facilitate cell adhesion, proliferation, and morphogenesis were obtained by the electrospinning method by Zhou et al. [121]. For such scaffolds, the homogeneity of nanofiber distribution was achieved by the incorporation of LAP in the polymer solution. Moreover, the viscosity and conductivity of the sample increase with the LAP concentration. Through mixing LAP with gellan gum, nanocomposite bioinks with high stability were generated with adjustable swelling features and improved shape retention, compared with those without LAP [122]. Besides this, the LAP/gellan gum nanocomposites present a stiffer matrix that is able to retain cells in their scaffold network. The absorption/release properties of these nanocomposites were studied using model proteins (lysozyme, bovine serum albumin), and the results demonstrated their retention capability and the absence of burst release.

A new outlook for developing scaffolds, smart medical devices, sensors, biorobots, bioactuators, etc., is given by 4D printing technology [123,124]. This technology allows 3D printed products to evolve in time and change their shape and properties under the action of various stimuli (temperature, pH, humidity, stress, electricity, magnetic field, light, acoustics) and to become smart printable inks (with shape memory, self-adaptability, self-sensing, and other multiple functionalities) [124]. Guo et al. [123] obtained 4D printed hydrogels by in situ polymerization of acrylamide in an agarose matrix containing LAP. LAP gives thixotropic and shear thinning behavior to the hydrogels, improving the 4D printing process, making extrusion easier, and increasing the stability of the shape after printing. The thermal reversible sol–gel transition of agarose made the 4D hydrogel recover its mechanical properties, namely softening by heating and hardening by cooling.

#### 5.1.4. Biosensors

Biosensors are analytical devices able to convert the biological impulse into a signal (namely, an electrochemical signal, pH, heat, light, etc.) that can be quantified and processed. These devices, able to provide rapid and accurate results, are widely used in environmental monitoring, food safety industries, early detection and control of some diseases, detection of protein cancer biomarkers, etc. [125]. The ability to exchange cations gave LAP the opportunity to serve as a matrix for the transfer of electroactive ions. Among the first papers that report the use of LAP in the manufacturing of biosensors refers to the development of a glucose biosensor [126]. This biosensor consists of an enzyme entrapped in a matrix of LAP gel by crosslinking with glutaraldehyde. By using the crosslinker, the properties of the sensor increase compared to those of pure LAP in terms of enzyme stability, sensor lifetime, and enzyme release. Recently, an electrochemical sensor based on an inkjet printed graphene electrode, modified with a thin film of LAP, was prepared for the detection of epinephrine (also called adrenaline) [127]. The obtained results show a good sensitivity for detecting epinephrine in aqueous solutions. The sensor achieved detection limits of 0.34 μM and 0.26 μM with an inkjet printed graphene electrode and with that modified, respectively. Considering that the deficiency or maladjustment of ascorbic acid (AA), uric acid (UA), and dopamine (DA) levels in the body can lead to severe diseases (such as cancer, Parkinson’s disease, cardiovascular disease, etc.), Ding et al. [128] propose a method to detect these compounds by using a LAP/graphene composite prepared by mechanical grinding of the two components. The electrocatalytic activity of graphene allows the composite to detect simultaneous AA, UA, and DA.

Oxalic acid (OA) is another organic acid with high solubility in water, present in spinach, cruciferous vegetables (such as cabbage, Brussels sprouts, and broccoli), and mushrooms. In the body, when it exceeds certain limits, it removes calcium from the blood and causes disorders in the activity of the heart, the nervous system, the digestive tube, and the kidneys. Therefore, there was a need to obtain sensors to detect its level in biofluids [129,130,131,132]. Joshi et al. [133] proposed an electrochemical biosensor used in the determination of OA, built by the deposition of LAP and ionic liquid (1-ethyl-3-methyl imidazolium chloride) film on a glass electrode (indium tin oxide). Due to its construction, this biosensor presents some advantages. On the one hand, the ionic liquid brings stability to the LAP film and a conductivity better than pure LAP; on the other hand, although it is devoid of enzymes, it can detect OA. The maximum sensitivity of the biosensor for this organic acid ranged from 1 mM to 20 mM.

**Figure 14:**
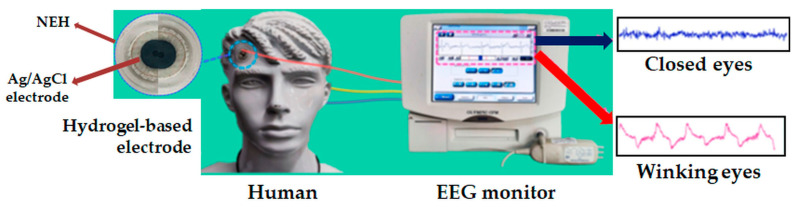
Illustration of the acquisition of bioelectrical signals by the nanoclay-enhanced hydrogel electrode (Adapted from [134]. CC BY 4.0 license).

A new hydrogel containing LAP for wet electrodes has been developed by incorporating LAP into a precursor solution containing acrylamide, N,N-methylenebisacrylamide, ammonium persulfate, NaCl, and glycerin and then thermo-polymerizing it at 40 °C for 2 h [134]. The hydrogel has great mechanical properties, with a tensile strength of 93 kPa and a breaking elongation of 1326%. It also sticks very well, with an adhesive force of 14 kPa, due to its double crosslinked network and nanoclay inclusion. Additionally, glycerin ensures excellent long-term stability of electrophysiology signals. The electrode based on this hydrogel can be applied as a wearable self-adhesive monitor to sensitively and stably acquire electrocardiogram and electroencephalography signals from the human body over extended periods (Figure 14) [134].

To monitor phenol, which has negative effects on the environment due to its toxicity and persistence, an amperometric biosensor was designed by trapping a polyphenol oxidase in a nanocomposite film containing LAP and CS [135]. The electrostatic interaction between positively charged CS chains and negatively charged LAP nanoparticles led to an increase in the biocompatibility, mechanical resistance, and adhesion of the nanocomposite to glass carbon electrodes. The biosensor exhibited a good affinity to its substrate, high sensitivity (674 mA/M/cm^2^) for catechol), and remarkable long-term stability in storage (it retains 88% of the initial activity after 60 days). The efficiency of biosensors can be enhanced by optimizing the composition and thickness of CS/LAP/polyphenol oxidase deposition.

#### 5.1.5. Biomedical Imaging

Properties such as tunable morphology, stability in biological environments, and a large active surface able to efficiently load imaging agents have allowed LAP to become a promising candidate for improving imaging contrast and sensitivity in biomedical imaging modalities [136]. For T1-weighted MR imaging applications, dendrimer–functionalized LAP nanodisks loaded with gadolinium were developed by Mustafa et al. [137]. Cell viability assays demonstrate noncytotoxicity, while the nanocomplexes exhibit high r_1_ relaxivity (2.05 1/(mM·s)), making them efficient contrast agents for T1-weighted MR imaging both in vitro and in vivo. These nanocomplexes show promise as a versatile platform for MR imaging in various biological systems.

Zhuang et al. [138] developed a hybrid nanoplatform using LAP nanodisks and polyethylenimine (PEI) for targeted computed tomography (CT) imaging and chemotherapy of CD44-overexpressed tumors. The platform, assembled with poly(lactic acid)–poly(ethylene glycol) (PLA–PEG–COOH) and gold nanoparticles, loaded with DOX, and modified with hyaluronic acid, demonstrated high stability, efficient drug loading, and pH sensitive release. In vitro and in vivo studies confirmed specific delivery to cancer cells, tumor growth inhibition, reduced DOX side effects, and effective CT imaging. This nanoplatform shows promise for targeted imaging and chemotherapy of CD44-overexpressed tumors.

An LAP-based magnetic resonance imaging agent was prepared for cancer cells overexpressing folate receptors by Ding and coworkers [139]. The preparation of this agent involves several steps: (i) The synthesis of LAP stabilized Fe_3_O_4_ NPs by controlled coprecipitation; (ii) PLA–PEG–COOH assembly on the surface of LAP/Fe_3_O_4_ NPs to provide additional stability; (iii) The conjugation of folic acid-modified generation 2 polyamidoamine dendrimer (G2–FA) using EDC coupling chemistry. The resulting LAP/Fe_3_O_4_–PLA–PEG–G2–FA nanoparticles exhibited excellent colloidal stability, biocompatibility, and enhanced r_2_ relaxivity (327.6 1/(mM·s) for MR imaging. It has been demonstrated that specific targeting of cancer cells involves high folate receptor expression and efficient uptake via folate–mediated endocytosis. In vivo experiments confirmed the efficacy of LAP/Fe_3_O_4_-based imaging in targeted MR imaging of folate receptor–overexpressing cancer cells.

A polydopamine-coated LAP nanoplatform was developed for photoacoustic (PA) imaging-guided chemo-phototherapy of integrin α_v_β_3_-overexpressed cancers [140]. This platform efficiently loaded the photosensitizer indocyanine green and the anticancer drug doxorubicin (DOX) separately, with poly(ethylene glycol)–arginine–glycine–aspartic acid modified on the surface as targeting agents. The resulting nanoplatforms demonstrated good colloidal stability, excellent photothermal and PA imaging properties, and pH sensitive, NIR-triggered DOX release. They were specifically taken up by 4T1 cells with integrin α_v_β_3_–overexpression, serving as targeted contrast agents for in vivo PA imaging. In vivo experiments revealed significantly stronger therapeutic effects and higher survival rates compared to monotherapy, showing synergistic chemo-phototherapy under NIR laser irradiation. This hybrid nanoplatform holds promise for PA imaging-guided chemotherapy of cancer cells overexpressing α_v_β_3_ integrin, hinting at future innovations in precise cancer theranostics.

Photo-induced cancer therapies, such as photothermal therapy (PTT) and photodynamic therapy (PDT), offer high selectivity and minimal side effects. However, they often require high laser power or photosensitizer dosages. To address this, a composite nanocarrier based on LAP–polypyrrole nanodisks was synthesized, offering excellent stability and biocompatibility [141]. Coating with poly(vinylpyrrolidone) enhances colloidal stability. The loading capacity for Chlorin e6 was exceptionally high (89.2%). In vitro and in vivo studies confirmed the combined PTT and PDT effect of nanocarriers, effectively suppressing tumor proliferation.

### 5.2. Food Packaging

Considering the recent implementation of environmental regulations and health considerations, food packaging research aims to incorporate LAP clay into the matrix of (bio)polymers (polysaccharides, proteins, and ecopolymers) in order to improve the thermal, mechanical, barrier, and antioxidant properties of (bio)nanocomposites films [142,143,144,145]. Valencia et al. [146] developed nanocomposite films based on gelatin with improved mechanical properties by adding LAP without affecting the optical, thermal, moisture content, water vapor permeability, or cristallinity properties. The interactions between the clay and the OH groups of the polymer chains in the matrix become stronger with the increase in clay concentration, improving the water vapor barrier and water resistance properties of starch/PVA/LAP-based bionanocomposite films [147]. Moreover, the results show improved mechanical properties up to 10% LAP content due to enhanced interface interactions. Olivera et al. [148] use as a plasticizer a mixture of lactic acid, glycerine, and PEG (combination ratio of 1:1:1) in the preparation of kafirin-based nanocomposite films with different concentrations of LAP. The films with a low content of LAP (3%) showed better water vapor permeability than those without LAP or with a high content of clay (5% and 10%). For the nanocomposite with 3% LAP, the clay nanoplatelets are completely separated and dispersed uniformly in the kafirin matrix, increasing the diffusion path length for water vapor. As the clay concentration increases, the surface of the film becomes more hydrophobic and opalescent and has high barrier properties. The current interest of researchers in the food packaging field is to find new materials that extend the food preservation capacity of the packaging, increase the shelf life of the food, improve its sensory quality, or make it edible. Starting from the positive results of onion-based materials on sensory properties, quality, and extended shelf life of beef hamburgers [149], Barbosa et al. [150] designed the onion/LAP composite films with properties able to extend the shelf life of food products. The appearance of crystalline domains, especially for LAP contents of 5% and 10%, respectively, increases the degree of opacity and hydrophobicity of the surface without changing the permeability to water vapor. Moreover, the onion/LAP composite films present good antioxidant properties, given both the onion’s high content of flavonoids and anthocyanins as well as the high concentrations of LAP. The antioxidant activity was also noted for gelatin films with high LAP content [151]. Aggregation of clay nanoplatelets (oriented parallel to each other or interspersed) in small domains, favored by high LAP concentrations, makes the migration path of gases (especially oxygen) and water vapor in the polymer matrix sinuous. In this way, the oxidation of lipids and the decomposition of meat proteins during storage are prevented, which means a longer shelf life for the products [152,153].

### 5.3. Drilling Fluids (DF)

**Figure 15:**
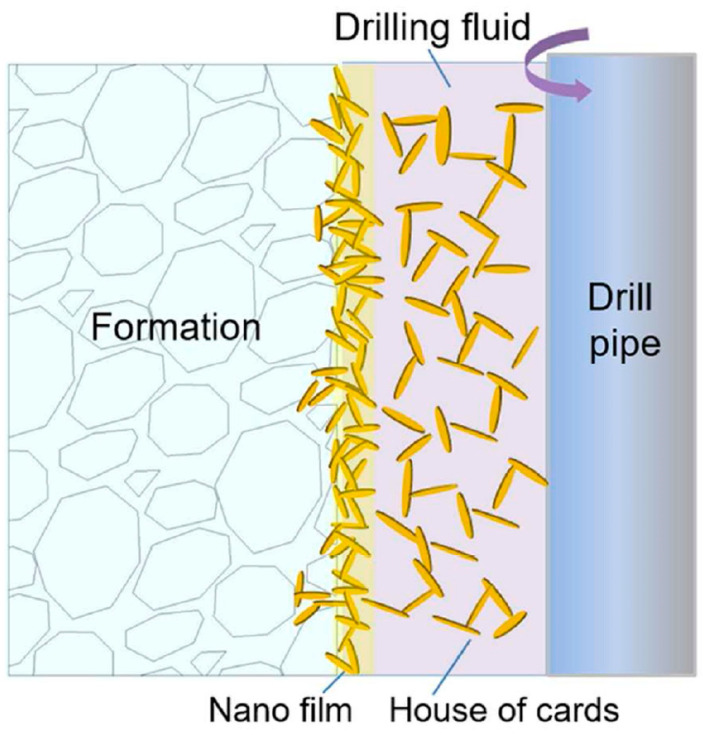
Illustration of shale pore plugging mechanism of LAP suspension (Adapted with permission from [170]. Copyright © 2018 American Chemical Society).

LAP is an important component in drilling fluids utilized in the oil and gas industry [154]. It exhibits superior shale inhibition, plugging properties, and lubrication enhancement in wellbore fluids. Additionally, it increases apparent viscosity without significantly affecting plastic viscosity, making it a promising inorganic viscosifier with the potential to reduce fluid loss [155]. In the fields of oil and gas, tailoring DF formulations that can support the drilling process in different conditions and avoid potential problems that may occur during drilling is of great importance [156,157,158,159]. Therefore, the DF must present some properties (such as pseudoplasticity, thixotropy, yield stress, etc.) to ensure the lubrication, cooling, and cleaning of the drill bit, the stability of the well, the formation of the filter cake on the permeable wells, the suspension and mobility of the drilling cuttings to the surface from the underground to the surface, etc. [160,161]. Mainly, DF (also called mud drilling) is composed of the following: (i) A liquid phase, which can be water with or without salt content (DF are called water-based muds), oil (DF are called oil-based muds), or emulsion; (ii) A solid phase that includes clay, cuttings, and weighting agents; (iii) Additives (inorganic, organic, or polymer compounds) [157,162]. Montmorillonite [163,164] and clays of the argillosepiolite/palygorskite group [165,166,167] are commonly used in DF, but synthetic silicate clays are also increasingly used due to their properties. LAP, sodium magnesium silicate synthetic clay, stands out for its good suspension, dispersion, thickening, and thixotropic properties [10,168]. It is able to control the viscosity, fluid loss, and cutting suspension during drilling operations, improving wellbore stability, hole cleaning efficiency, and providing lubrication to drilling equipment [22,169,170]. The use of LAP in a drilling fluid can be beneficial for sealing shale pores. When there is a positive differential pressure between the drilling fluid (with high pressure) and the formation (with low pressure), the LAP particles are pushed toward the wellbore wall, thereby obstructing the shale pores (Figure 15) [170].

The rheological properties of LAP/polyionic cellulose dispersion recommend the use of LAP (of concentration 1%) in the development of drilling fluid formulations for wellbore cleaning and cuttings circulation in high-temperature horizontal wells over bentonite (of concentration 4%) [171]. Although both clays induced pseudoplastic properties in the polyanionic cellulose solution, the formation of the “star network” between LAP and polyanionic cellulose solution (both in the absence/presence of temperature) led to a more structured gel, a high value of yield stress, and a fast recovery rate. In horizontal drilling in shale gas pockets, the contact area between the drill pipe and the wellbore is larger than in the case of vertical drilling, causing a series of problems (such as pipe blockage). For this purpose, Huang et al. [155] found that LAP can cause shale inhibition better than polyether amine and KCl.

In addition, LAP can improve the lubrication properties of the DF and reduce the contact between the drill pipe and the wellbore, as well as fluid losses. As the depth of the borehole increases, high temperatures (exceeding 200–240 °C [22,23], high pressure, or salt intrusions (when drilling through salt beds) [172] damage the properties of drilling fluids (e.g., viscosity, filtration properties, etc.) as they are exposed to this environment for a long time [22,23,158,172]. Shen et al. [172] developed a nanocomposite in which the nontoxicity, temperature, and salt resistance abilities of hydrophilic-associated polymers were combined with the structural properties of LAP, giving a water-based DF with improved thermal stability, salt tolerance, and fluid loss control.

The investigation of poly(ethylene glycol)/LAP and poly(propylene glycol)/LAP dispersions with more than 2.55% and 3.10% clay, respectively, could be considered for drilling fluid formulation [10]. The addition of NaCl increased the yield point value of dispersions while decreasing their plastic viscosity, rendering them suitable for drilling fluid applications. Furthermore, with the addition of NaCl, the complex viscosity of these dispersions exhibited temperature independence within the range of 10–65 °C. Additionally, the zeta potential value decreased with increasing LAP concentration up to approximately 3% clay, beyond which higher clay content had no significant effect [10].

The new LAP/isopentenol polyoxyethylene ether composite enhances the rheological properties of water-based drilling fluids under various conditions, including before and after aging at 240 °C, and it demonstrates resistance to 15% NaCl [173]. In water-based drilling fluids, the combination of LAP, isopentenol polyoxyethylene ether, and bentonite creates a flexible and robust network, allowing for better suspension and efficient transport of debris. Moreover, this composite ensures a more efficient cleaning of the wellbore, especially in environments with extremely high temperatures and salinities.

A high-stability gel was developed using acrylamide as the monomer, a self-synthesized microsphere containing active ethylene groups as the crosslinker, LAP as a flow regulator and filler, and ammonium persulfate as the initiator [174]. The high-stability polymer gel offers effective control of drilling fluid loss in fractured formations, even under conditions of high temperature and salinity.

A modified LAP additive was prepared using the surface sol–gel technique, where perfluorohexylethyltrimethoxysilane was deposited onto the surface of LAP particles [175]. Modified LAP additives, characterized by lyophobic wettability, contribute to the stability of emulsions, improve rheological properties, and strengthen wellbore integrity. It forms a sheet-like structure compatible with shale pore sizes and withstands temperatures up to 450 °C.

In response to growing environmental concerns, there has been an increased focus on developing environmentally friendly and high–performance water-based drilling fluids (WBDFs) to replace traditional oil-based drilling fluids. However, many existing WBDFs still rely on synthetic materials, limiting their environmental benefits. To address this, a novel environmentally friendly WBDF (EF–WBDF) was developed using LAP nanoparticles and derivatives of natural materials such as crosslinked starch, cellulose composite, gelatin ammonium salt, poly–l–arginine, and polyanionic cellulose [176]. This fluid proves thermally resilient up to 150 °C, maintains rheological consistency, and shows potential for high-temperature wells, making it a viable option for drilling in sensitive and high-temperature regions.

Dong and coworkers [177] developed a nanocomposite filter reducer (ANDP) for drilling fluids in deep formations using a blend of 2-acrylamide-2-methylpropane sulfonic acid, acrylamide, and modified nanoLAP. ANDP demonstrates excellent resistance to high temperatures and salinity, ensuring stable drilling fluid performance. Analyzed using electron microscopy and spectrometry, ANDP shows robust thermal stability and proper molecular formation. ANDP enhances the hydration layer of clay particles, preserving the fluid’s colloidal stability and preventing particle clumping, resulting in a denser mud cake and reduced filtration.

### 5.4. Cosmetics and Personal Care Products

It is known that clays, including LAP, represent a versatile ingredient that enhances product stability, texture, and performance in cosmetics and personal care items [178,179,180]. LAP was used as a co-binder with sodium carboxymethyl cellulose in the formulation of stable toothpaste [181]. The inclusion of the LAP/sodium carboxymethyl cellulose mixture in the toothpaste determines the increase in thixotropy and shear thinning in the paste, making extrusion easier. The presence of electric charges in smectites, such as LAP, allows for ion exchange capacities beneficial for loading active cosmetics and adsorbing waste substances. In polar media, smectites with negative charges undergo layer expansion, imparting specific rheological properties valuable in cosmetic formulations [182].

Stable particles with antimicrobial properties, which use a small amount of silver particles loaded onto LAP platelets and have possible applicability in the preparation of cosmetic products, were designed by Disalvo and Mordas [183]. The introduction of LAP as an ingredient in cosmetic formulations confers the product with a light, nonsticky texture, improving the feeling of the skin due to its fine particle size. The hydrogels based on LAP and PEO with low cytotoxicity for use in personal care and cosmetic products were developed by Schmidt and coworkers [184,185]. Smectite clays, including LAP, are used in the formulation of cosmetic skin protection products due to their high sorption capacity [186].

A selection of more recent works on LAP-based materials with their possible applications is presented in Table 3.

Researchers continue to develop new formulations with LAP due to the versatility and advantages that this clay has, although it is already incorporated in a variety of commercial products (https://www.byk.com/en, accessed on 22 May 2024): (i) In facial masks and skincare products where LAP improves the texture and stability of their formulations; (ii) In household cleaning products where LAP ensures the distribution and suspension of cleaning agents; (iii) In hair and body care products where LAP ensures a specific viscosity; (iv) In paints and coatings where LAP improves paint consistency and drying properties; (v) In cleaning gels for art conservation, etc. Moreover, for materials in biomedical applications, LAP is often mentioned in patents.

## 6. Conclusions

The exceptional properties of LAP give it an advantage over other compounds and make it a suitable candidate in the design of new innovative materials. The structure and layered nature of LAP in water are responsible for the diversity of the phase states (liquid, sol, gel, glass, and flocculation) induced by factors such as ionic strength, polymer addition, time, pH, etc.

Knowing and understanding the way in which these factors affect the structure of the LAP dispersion allows the selection of the appropriate conditions for obtaining a material with properties tailored for specific applications. The multifaceted properties of LAP make it a versatile material with diverse applications across various fields. In biomedicine, its biocompatibility, nontoxicity, and osteoinductive properties hold promise for drug delivery systems, tissue engineering scaffolds, and orthopedic implants, aiming to improve patient care and outcomes. Additionally, its ability to form a gel in aqueous dispersion finds applications in wound healing materials. LAP serves as a stabilizer and thickening agent in the food industry, and it also acts as a rheology modifier, enhancing drilling efficacy and stability in drilling fluids due to its gel-forming capacity. Furthermore, the presence of LAP in cosmetic formulations provides stability and viscosity control.

## 7. Future Trends

The versatile properties of LAP pave the way for further opportunities and perspectives, spanning from advanced sensors and antiviral materials to dynamic 4D printing applications, offering innovative solutions across biomedical, environmental, and engineering domains.

Research could focus on developing multifunctional sensors that incorporate LAP nanoparticles and are able to detect both environmental pollutants and viral pathogens. By integrating specific receptors or antibodies onto the LAP surface, these sensors could offer rapid and sensitive detection of viral antigens or RNA, enabling early diagnosis of viral infections in clinical settings and public health surveillance. The design of new biosensors based on LAP, which can identify small molecules such as cholesterol, glucose, and urea in their early stages, represents a challenge for researchers. LAP could be explored as a versatile platform for developing biosensing technologies, leveraging its unique properties, such as its high surface area, biocompatibility, and ability to interact with biomolecules. By immobilizing biomolecules or enzymes onto the LAP surface, researchers could create sensitive and selective biosensors for detecting a wide range of analytes, including biomarkers, toxins, and pathogens, with potential applications in healthcare diagnostics, food safety, and environmental monitoring.

The integration of LAP-based materials into theranostic systems could enable simultaneous detection and treatment of viral infections. By combining diagnostic functionalities, such as viral detection sensors, with therapeutic components, such as drug-loaded LAP nanoparticles, researchers could create advanced theranostic platforms capable of personalized and targeted management of viral diseases, thus improving patient outcomes and reducing healthcare costs.

Exploration of LAP-based nanocomposites could lead to the development of versatile antiviral materials with applications in healthcare, consumer products, and public spaces. By combining LAP with antiviral agents, such as silver nanoparticles or antiviral peptides, researchers could create materials capable of actively inhibiting viral attachment, replication, and transmission on various surfaces, including textiles, plastics, and coatings. In the wake of recent global health crises, LAP has also emerged as a promising material for the development of antiviral coatings and filters, providing protection against airborne pathogens and viruses [188]. Its ability to immobilize and deactivate viruses on surfaces offers a potential solution for mitigating the spread of infectious diseases in various settings, including healthcare facilities, public spaces, and transportation systems.

Further research into 4D printing materials containing LAP could focus on enhancing their responsiveness to external stimuli, such as temperature, pH, or light. By optimizing the composition and structure of LAP-based composites, researchers could design dynamic materials capable of undergoing complex shape transformations or functional changes in a controlled manner, opening up new possibilities for applications in soft robotics, biomedical devices, and adaptive structures.

Laponite^®^ remains a promising nanomaterial that deserves further investigation due to its low cost and its special properties, which allow it to be included in materials usable in a wide range of applications.

## Figures and Tables

**Table 1:** Comparative table with the chemical composition, physical properties, applications, and key differences between the main LAP types (https://www.byk.com/en, accessed on 22 May 2024; https://www.matweb.com/, accessed on 23 May 2024).

LAP Type	Composition	Physical Properties	Applications	Differences
RD	SiO_2_: 59.5% MgO: 27.5% Na_2_O: 2.8% Li_2_O: 0.8% Loss on ignition: 8.2%	bulk density: ~1.0 g/cm^3^ specific surface area: ~370 m^2^/g pH (2% dispersion): 9.8	- rheology modifier in coatings and paints; - cosmetics (thickening agent); - pharmaceuticals; - adhesives.	- standard grade; - widely used for its high purity and consistency.
RDS	SiO_2_: 54.5% MgO: 26% Na_2_O: 5.6% P_2_O_5_: 4.4% Li_2_O: 0.8% Loss on ignition: 8%	bulk density: ~1.0 g/cm^3^ specific surface area: ~330 m^2^/g pH (2% dispersion): 9.7	- personal care (lotions, gels); - household products (detergents); - surface coatings; - industrial suspensions.	similar to RD, with slight differences in surface area and viscosity for specific applications.
XLG	SiO_2_: 59.5% MgO: 27.5% Na_2_O: 2.8% Li_2_O: 0.8% Loss on ignition: 7%	bulk density: ~1.0 g/cm^3^ specific surface area: ~370 m^2^/g pH (2% dispersion): 9.8	- research (model colloidal systems); - pharmaceuticals; - personal care products; - paints and coatings.	- lower specific surface area; - used in applications requiring lower viscosity.
XLS	SiO_2_: 54.5% MgO: 26% Na_2_O: 5.6% P_2_O_5_: 4.1% Li_2_O: 0.8% Loss on ignition: 8.2%	bulk density: ~1.0 g/cm^3^ specific surface area: ~330 m^2^/g pH (2% dispersion): 9.7	- enhanced rheology for clear gels; - cosmetics; - personal care (transparent formulations); - coatings.	optimized for transparent formulations with lower turbidity and enhanced clarity.
JS	SiO_2_: 50.2% MgO: 22.2% Na_2_O: 7.5% P_2_O_5_: 5.4% Li_2_O: 0.8% Loss on ignition: 8.2%	bulk density: ~0.950 g/cm^3^ specific surface area: ~300 m^2^/g pH (2% dispersion): 10	- oil drilling fluids; - construction materials; - ceramics.	designed for industrial applications such as drilling fluids, where higher performance in suspension is needed.

**Table 2:** Advantages and disadvantages of LAP synthetic clay compared with natural clay.

	LAP	Natural Clay
Advantages	- uniform and controlled composition; - high purity; - well-defined nanometric particle size and shape; - can be tailored for specific applications; - excellent dispersion in aqueous and nonaqueous systems; - superior and controllable rheological properties; - ore regulated and controlled for specific applications;	- lower environmental impact; - less expensive and widely available;
Disadvantages	- synthetic production can have a higher environmental footprint; - more expensive due to manufacturing processes;	- composition can vary significantly depending on the source; - often contain impurities; - variable particle size and shape, which can impact performance; - limited ability to modify inherent properties; - can be difficult to disperse uniformly in some formulations; - rheological properties can vary and are less predictable; - can contain trace elements or contaminants; - cannot be easily modified for specific applications;

**Table 3:** Selection of relevant papers (reviews/research papers/patents) on LAP-based materials with various applications.

Field	Reviews	Application	Composition	Research Papers/Patents
Biomedical Applications	[83,84,85] [111,114] [116,118]	Drug delivery	LAP/CS/PVA LAP/DNA/Oxidized Alginate LAP/cPEG/DOX LAP/Poly(acrylate)/Sodium phosphate LAP/Heparin/Poloxamer 407 LAP/quaternized CS/gelatin LAP/Antiviral Agents	[11] [91] [94] [100] [101] [187] [188]
		Tissue engineering	LAP/Sodium Alginate LAP/CS LAP/PEGDA	[106] [112] [113]
		Bioprinting	LAP/Stromal cells LAP/Caprolactona LAP/Gelan Gum LAP/PAAm/Agarose	[120] [121] [122] [123]
		Biosensors	LAP/Graphene Electrode LAP/PAAm	[127] [134]
		Biomedical imaging	LAP/Polyethylenimine LAP/Fe_3_O_4_ NP LAP/Polydopamine LAP/Polypyrrole	[138] [139] [140] [141]
Food Packaging	[142,143] [144,145]	Food properties (i.e., sensory, quality, shell life, etc.) Packaging properties	LAP/Gelatin LAP/(Lactic Acid/Glycerine/PEG) mixture (1:1:1) LAP/Onion LAP/Gelatin LAP/Pectin/Ag NP	[146] [148] [150] [151] [153]
Drilling fluids	[157,161]	Drilling operations Lubrication of drilling equipment Wellbore integrity Cleaning hole	LAP/Polymer Nanocomposites LAP/Polyionic Cellulose LAP/PEG; LAP/PPG LAP/Isopentenol Polyoxyethylene Ether LAP/Perfluorohexylethyltrimethoxysilane LAP/Polysaccharide/Polypeptide	[156,157,158,159,172,177] [171] [10] [173] [175] [176]
Cosmetics and Personal Care Products	[180]	Emulsifying, thickening, suspending, anticaking and moisturizing agent	LAP/Sunscreen Formulation	[179]

## Data Availability

No new data were created or analyzed in this study.

## Abbreviations

AA	ascorbic acid
AAm	acrylamide
AMPS	2-acrylamido-2-methylpropanesulfonic acid
ANDP	nanocomposite filter reducer based on 2-acrylamide-2-methylpropane sulfonic acid, acrylamide, and modified Laponite^®^
c*	overlap concentration of poly(ethylene oxide)
c_LAP_	Laponite^®^ concentration
c_sat_	critical polymer concentration corresponding to a complete cover of the clay platelet surface
CB[6]	curcubit[6]uril
cPEG	cyclic poly(ethylene glycol)
CS	chitosan
DA	dopamine
DF	drilling fluids
DNA	deoxyribonucleic acid
DOX	doxorubicin
FFA	flufenamic acid
HP copolymer	heparin–poloxamer 407
LAP	Laponite^®^
MYR	isopropyl myristate
M_w_	polymer molecular weight
OA	oxalic acid
PAA	poly(acrylic acid)
PAAm	poly(acrylamide)
PDT	photodynamic therapy
PEG	poly(ethylene glycol)
PEGDA	poly(ethylene glycol) diacrylate
PEO	poly(ethylene oxide)
PLA–PEG–COOH	poly(lactic acid)–poly(ethylene glycol)
PPG	poly(propylene glycol)
PTT	photothermal therapy
PVA	poly(vinyl alcohol)
PZC	point of zero charge
SQ	squalene
t_w_	aging time
UA	uric acid
WBDF	water-based drilling fluid
